# Revisiting Caregiver Preparedness: Development of a Multidimensional Measure for Dementia Caregivers

**DOI:** 10.1002/alz70858_103516

**Published:** 2025-12-26

**Authors:** Emily Mroz, Kristie Wood, Stephani Shivers, Marney White, Joan K. Monin

**Affiliations:** ^1^ Emory University, Atlanta, GA, USA; ^2^ University of Colorado, Anschutz Medical School, Aurora, CO, USA; ^3^ CaringKind, New York, NY, USA; ^4^ Yale School of Public Health, New Haven, CT, USA

## Abstract

**Background:**

Many of the over 11 million adults serving as family caregivers to people living with dementia feel *unprepared* for their caregiving roles. Lack of preparedness for caregiving has major consequences for health and decision‐making outcomes for caregivers, their care recipients, and family networks, but the limited number of measurement options have stymied research in this area. The only widely used caregiver preparedness measure was developed over 30 years ago. That measure is unidimensional, focuses on skill‐based competencies, and is not specific to dementia‐caregiving. Improving the measurement of preparedness for dementia‐caregiving is critical for identifying caregivers who are most in need of early interventions and for developing tailored support that addresses specific preparedness needs.

**Method:**

We developed the Dementia‐Caregiving PRactical Evaluation of Preparedness (PREP). Our process was guided by a panel of caregiving thought leaders. We will describe: a) concept mapping to develop three preparedness dimensions (i.e., confidence and psychological readiness, skills and insights, personal circumstances and related capacity), b) member‐checking with a sample of 66 dementia‐caregivers and care professionals via online survey, and cognitive debriefing via online semi‐structured interviews with eight dementia‐caregivers and care professionals.

**Result:**

Member checking participants rated the ease of answering each item (*M* = 3.34/4), how well the item fit within the domain it was grouped (*M* = 3.76/4), and how relevant the item was to the overall concept of preparedness (*M* = 3.81/4) on a scale from 1 = disagree to 4 = agree. Scores demonstrated the quality of the candidate items and the relevance of items across the three dimensions of dementia‐caregiver preparedness. Item scores, survey feedback, and cognitive debriefing were used to reduce the pool of items to a final set of 30 items.

**Conclusion:**

This study demonstrates the value of a multidimensional approach to measuring dementia‐caregiving preparedness: feedback reinforced that preparedness for dementia‐caregiving can vary across three dimensions. The Dementia‐Caregiving PREP measure will be distributed to conference attendees and can be used to examine associations between caregiver characteristics and caregiving outcomes. This research will improve early intervention efforts to support caregiver and care recipient wellbeing.

